# Characterization of the lncRNA-miRNA-mRNA regulatory network to reveal potential functional competing endogenous RNAs in traumatic brain injury

**DOI:** 10.3389/fnins.2022.1089857

**Published:** 2023-01-11

**Authors:** Jiangtao Yu, Zijun Lu, Ruining Liu, Pengcheng Wang, Haoli Ma, Yan Zhao

**Affiliations:** ^1^Emergency Center, Zhongnan Hospital of Wuhan University, Wuhan, Hubei, China; ^2^Department of Biological Repositories, Zhongnan Hospital of Wuhan University, Wuhan, China; ^3^Hubei Clinical Research Center for Emergency and Resuscitation, Zhongnan Hospital of Wuhan University, Wuhan, Hubei, China

**Keywords:** traumatic brain injury, RNA-seq, lncRNA, miRNA, ceRNA network

## Abstract

Traumatic brain injury (TBI) is one of the most common acute central nervous system injury diseases. Given the medical and socio-economic burdens of TBI patients, the pathogenesis in TBI and the latent intervention targets needed to be further illuminated. Long non-coding RNAs (lncRNAs) had been revealed to play a vital role in the regulation of pathogenesis after TBI. However, the mutual communication and adjustment of lncRNA associated competing for endogenous RNA (ceRNA) networks in TBI have not been explored to date. In this study, we systematically sequenced the whole transcriptome of lncRNAs, miRNAs, and mRNAs between sham and TBI groups and a total of 939 differentially expressed (DE) lncRNAs, 46 DE miRNAs, and 1,951 DE mRNAs were obtained. Gene ontology (GO), Kyoto Encyclopedia of Genes and Genomes (KEGG) pathway, and protein interaction relationship analyses were conducted for DE mRNAs to identify hub DE genes in TBI. Based on the criteria of bioinformatics prediction, the lncRNA associated ceRNA network covering 201 lncRNAs, 22 miRNAs, and 79 mRNAs was constructed. This study provides a novel perspective on the molecular mechanism of lncRNA in TBI and identifies certain lncRNAs as potential therapeutic targets against TBI.

## Introduction

Traumatic brain injury (TBI) refers to brain function or pathological changes caused by trauma (Menon et al., 2010). TBI begins with an initial injury caused by mechanical forces supervening with a secondary injury due to a complicated bundle of cellular processes and biochemical cascades that occurs in minutes to days after the primary trauma. A large number of patients die from TBI each year, and many patients are temporarily or permanently disabled (Zaloshnja et al., 2008; Peeters et al., 2015). In 2013, approximately 2.5 million patients were treated in the emergency department for TBI in the USA, including 282,000 hospitalizations and 56,000 deaths (Taylor et al., 2017). From 2006 to 2013, the mortality rate of TBI in China remained high and the survivors after TBI with varying degrees of disability had a poor quality of life (Cheng et al., 2017). Furthermore, convulsions, strokes, and nervous system infections are common as neurological complications of TBI (Wang et al., 2019). It can be seen that TBI is a serious public health problem worldwide. Given the medical and socio-economic burdens of TBI patients, the pathogenesis in TBI and latent intervention targets needed to be further illuminated.

With the rapid development of omics technology such as genomics, transcriptomics, and proteomics, genetic events were found to exert crucial roles in TBI. A growing body of researches had indicated that non-coding RNAs (ncRNAs) were key regulators of neurological diseases and were attractive to control post-injury brain damage efficiently (Chandran et al., 2017). Bioinformatics analysis of the co-expression networks of mRNAs and lncRNAs altered after TBI showed that a majority of them were associated with inflammatory and immunological activity, metabolism, neuronal and vascular networks, and cellular function (Zhong et al., 2016). In fact, the previous study identified that the dysregulated lncRNAs might be related to the physiological and pathological processes after TBI (Chandran et al., 2017). For example, it was revealed that the up-regulated lncRNA *NEAT1* could inhibit apoptosis and inflammation, thereby resulting in better functional recovery in mice after TBI (Zhong et al., 2017). Meanwhile, the lncRNA *MALAT1* in exosomes drove regenerative function and modulated inflammation-linked networks following TBI (Zhong et al., 2017). Moreover, miRNAs also had been reported to be involved in the regulation of TBI by inhibiting effects on the formation of secondary brain damage (Pan et al., 2017). For example, it was reported that *miR-21* inhibited apoptosis and promoted angiogenesis through regulating the expression of apoptosis- and angiogenesis-related molecules after TBI in rats (Ge et al., 2014). It was also revealed that *miR-23b* improved cognitive impairments in TBI by targeting ATG12-mediated neuronal autophagy (Sun et al., 2018). Besides, it had been reported that the up-regulation of *miR-144* promoted cognitive impairments induced by β-amyloid accumulation post-TBI through suppressing of the *ADAM10* expression (Sun et al., 2017).

Competing endogenous RNA (ceRNA) networks had been found to be important mechanisms for affecting gene translation at the transcriptional level (Salmena et al., 2011). These ceRNAs included various RNA types such as the lncRNA-miRNA-mRNA network that lncRNA competed for miRNA to regulate mRNA and the potential regulatory pathways could be based on the shared bridge miRNA through miRNA response elements (MREs) (Fatica and Bozzoni, 2014). For example, previous study suggested that the lncRNA *MALAT1* induced apoptosis in the Parkinson’s disease (PD) model by sponging endogenous miR-124 (Liu et al., 2017). An *lnc-SCA7*/*miR-124*/*ATXN7* ceRNA network was identified to mediate the post-transcriptional crosstalk between *lnc-SCA7* and *ATXN7* mRNA and thus created a loop in *Spinocerebellar ataxia type 7* (*SCA7*) (Tan et al., 2014). Of course, there has been a lot of researches on ceRNAs in other different neurological diseases, such as Alzheimer’s disease (AD) (Roberts et al., 1652), ischemic stroke (Zhang et al., 2018), and spinal cord injury (SCI) (Gu et al., 2017). Previous studies have reported the changes of lncRNA and mRNA in TBI mice (Zhong et al., 2016), as well as the changes of miRNA in rat hippocampus after TBI (Hu et al., 2012) and ceRNA network of mice following TBI (Wang et al., 2022). Though these researchers have made a lot of contributions to TBI research, their research focused on the cortex of mice or the hippocampus of rats. The ceRNA networks in rat cerebral cortex after TBI have not been explored. Therefore, we are working to identify long non-coding RNAs (lncRNAs), microRNAs (miRNAs) and mRNAs that are differentially expressed (DE) in rat cerebral cortex after TBI, and thus to construct lncRNA-associated ceRNA networks and to reveal their potential mechanisms in TBI.

In this study, we established a controlled cortical impact (CCI) model to obtain TBI rats and used high-throughput sequencing to discover changes in lncRNAs, miRNAs, and mRNAs of rat cortex after TBI. Then the lncRNA-miRNA-mRNA ceRNA network was constructed by using the predicted interaction patterns between lncRNA and mRNA mediated by miRNA. And some genes were chosen to verify by using quantitative reverse transcription polymerase chain reaction (QRT-PCR). Our findings contribute to the ceRNA networks in underlying mechanisms of post-TBI physiological and pathological processes and unearth new targets that are particularly important for the development of TBI therapies.

## Materials and methods

### Controlled cortical impact model for TBI rats

Adult male Sprague-Dawley rats (weight 250–300 g) were purchased from Vital River Laboratory Animal Technology Co. Ltd. (Beijing, China). The CCI method was chosen to construct our TBI model which was established in rats as described previously (Smith et al., 1995). Sixteen rats were anesthetized by intraperitoneal injection of 5% pentobarbital at a dose of 50 mg/kg and their heads were fixed on a standard rodent stereotaxic frame after anesthesia. The skull was removed without hurting the dura mater until the notch diameter reaches about 5 mm. Eight rats were restrained by stereotaxic device and were subjected to the CCI device (Impact One™, Stereotactic Impactor for CCI, Leica Microsystem, IL, USA) with a 3 mm impactor tip was placed in the center of the craniotomy site and a moderate injury was induced with 5 m/s speed, 200 msec dwell time and 2 mm deformation depth, which resulted in a major focal injury of the right cerebral cortex. These actions induced moderate injury to the rat brains. The sham group (eight rats) received the same surgery but without actual injury by the impactor. After 24 h of survival, rats were fully anesthetized by 5% pentobarbital at a dose of 50 mg/kg and were perfused with the heart with 50 mL of isotonic saline. Then, the cerebral cortex tissue around the focal lesion was quickly dissected and stored in liquid nitrogen.

### RNA sequencing for lncRNA, miRNA, and mRNA

Total RNA samples of six rats (three rats each in sham and TBI groups) were extracted using the mirVana miRNA Isolation Kit (Ambion, USA) following the manufacturer’s protocol. The RNA integrity was evaluated using the Agilent 2100 Bioanalyzer (Agilent Technologies, USA) and the samples with RNA Integrity Number (RIN) ≥7 were subjected to the subsequent analysis. The cDNA library covering lncRNA and mRNA was constructed using TruSeq Stranded Total RNA with Ribo-Zero Gold (Illumina, USA) according to the manufacturer’s instructions. And the library of miRNA was constructed by purifying gel fragment enriched with DNA reversely transcribed and amplified from small RNA. Then these libraries were sequenced on the Illumina sequencing platform (HiSeqTM 2500) and the paired-end reads were generated.

Raw reads generated during high-throughput sequencing were sequences in fastq format. Trimmomatic software was first used to get high-quality clean reads through removing adapter sequences and filtering out low-quality bases and low-quality reads (Bolger et al., 2014). Then, hisat2 software was used to align clean reads to the reference genome of rat and Stringtie software was used to assemble the reads into transcripts (Pertea et al., 2015). The known mRNA and lncRNA transcripts were selected by comparing the gene annotation information of the reference sequence produced by Cuffcompare software (Trapnell et al., 2012). The coding potential of other unknown transcripts above 200 bp was screened out by CPC, CNCI, Pfam, and PLEK to obtain predicted lncRNA sequences, respectively. The gene quantitative analysis of lncRNA and mRNA was performed by using eXpress function of bowtie2 to obtain the FPKM values for each gene. Moreover, the known miRNAs were identified by aligning against the miRBase v.21 database,^1^ and the unannotated small RNAs were analyzed by mirdeep2 to predict novel miRNAs (Friedlander et al., 2012).

### Differential expression analysis

The estimateSizeFactors function of the DESeq R package was used to normalize the counts, and the nbinomTest function was used to calculate *p*-value and fold change values for the difference comparison. DE transcripts with *p*-value ≤ 0.05 and fold change ≥2 were selected, and these DE lncRNAs, miRNAs, and mRNAs between sham and TBI groups were identified, respectively. Hierarchical clustering was performed to show the distinguishable expression pattern among samples. Moreover, the heatmap was built by using the pheatmap R package.

### Gene ontology analysis and pathway analysis

The biological processes (BP) in Gene Ontology (GO) and the Kyoto Encyclopedia of Genes and Genomes (KEGG) were enriched by hypergeometric distribution tests to determine the biological functions or pathways that were primarily affected by differential genes. The GO categories were derived from GO,^2^ which comprise three categories: BP, cellular components (CC), and molecular functions (MF). Pathway analysis was provided, based on the latest version at KEGG website,^3^ which allowed us to determine the molecular interaction and reaction networks with the significantly changed mRNAs. The *p*-value (<0.05) denoted the significance of the pathway correlations.

### Protein-protein interaction network

The STRING online tool was used to obtain the protein-protein interaction relationship (PPI) of differential genes. After the PPI relationship obtained in the previous step, the Cytohubba plug-in of Cytoscape software was used to calculate the degree of connection of each node, and the important node of the PPI network participating in the protein interaction relationship, namely the hub protein, was obtained through its score ranking. Using the MCODE plug-in of Cytoscape software, the clustering analysis was applied to identify the functional modules in the PPI network and the relationships between the network topology and network components. The MCODE parameters were set as follows: Include Loops: false; Degree Cutoff: 10; Node Score Cutoff: 0.2; Haircut: true; Fluff: false; K-Core: 2; Max. Depth from Seed: 100.

### Construction of the ceRNA network

The intersections with the DE lncRNAs and mRNAs mediated by miRNA were analyzed. First, the miRNA-target (miRNA-lncRNA/miRNA-mRNA) pairs were predicted according to the base sequence by using the miRanda software. At the same time, the Pearson correlation coefficient and *p*-value of miRNA-target pairs were calculated based on the expression values of corresponding genes. Positively correlated miRNA-target (miRNA-lncRNA/miRNA-mRNA) pairs with a *p*-value < 0.05 and Pearson’s correlation coefficient >0.8 were chosen for further analysis. The intersections of the above two results could be used for the prediction of subsequent ceRNA networks. Finally, based on established co-expression data, the ceRNA networks about lncRNAs, miRNAs, and mRNAs were mapped using Cytoscape software.

### Quantitative RT-PCR

Total RNA samples were isolated from rat cerebral cortex from sham and TBI groups (five rats in each group) using TRIzol reagent (Invitrogen, USA). Before the reverse transcription reaction, total RNA sample (1 μg) was digested with RNase-free DNase I at 37°C for 30 min which was stopped by adding 1 μL EDTA (50 mM) at 65°C for 10 min to inactivate DNase I. Then, the mixture from the previous step was transferred to synthesize cDNA using the PrimeScript RT reagent Kit (Takara, Japan). Primer 3^4^ was used to design gene specific primer. The real-time qPCR reaction was performed using the SYBR Green assay (GenePharma, China) in a Light Cycler 480 real-time PCR system (Roche, USA) with the following conditions: 95°C, 10 min for one cycle; then 95°C, 30 s and 60°C, 30 s for 40 cycles. Beta-actin (*Actb*) was used as internal control and relative expression levels were calculated using the 2^–ΔΔ^*^Ct^* method.

### Statistical analysis

All data were analyzed using SPSS version 20.0 software (IBM Corp. Armonk, NY, USA) and presented as mean ± standard error of the mean (SEM). Student’s *t*-tests were used for All data were analyzed using SPSS version 20.0 software (IBM Corp. Armonk, NY, USA) and presented as mean ± standard error of the mean (SEM). Student’s *t*-tests were used for comparisons between two groups, whereas one-way analysis of variance was performed for repeated measures.

## Results

### Overview of lncRNA, miRNA, and mRNA sequencing in the cerebral cortex of TBI rats

**FIGURE 1:**
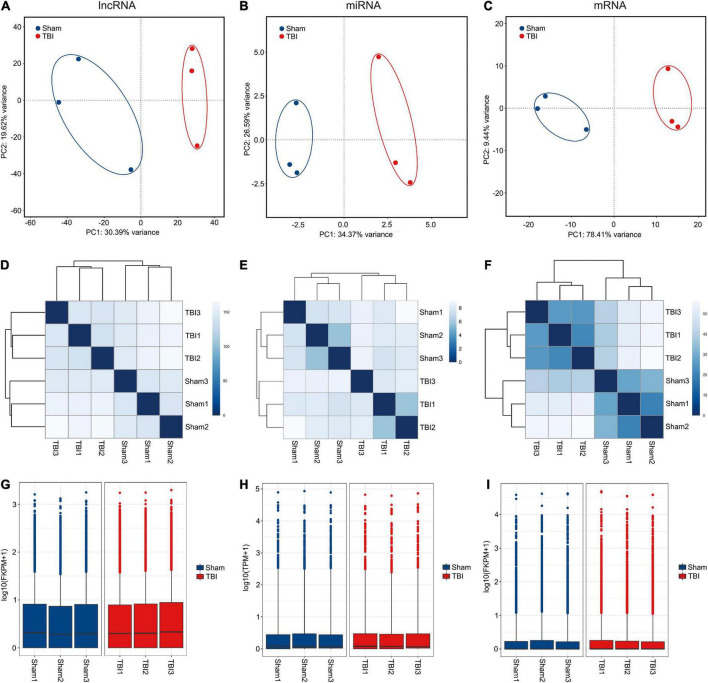
RNA sequencing of lncRNA, miRNA, and mRNA. **(A–C)** Principal component analyses of lncRNA, miRNA, and mRNA among samples from sham and TBI groups. The confidence ellipses are drawn to include samples from sham and TBI groups. **(D–F)** Sample-sample distances of lncRNA, miRNA, and mRNA among sham and TBI groups. **(G–I)** Box-whisker plots of lncRNA, miRNA, and mRNA among sham and TBI groups.

RNA sequencing experiments were performed to discover lncRNAs, miRNAs, and mRNAs related to the pathophysiological mechanism of TBI and to compare the transcriptional regulation of samples between sham and TBI groups. First, principal component analyses (PCA) were conducted on the expression level of lncRNAs, miRNAs, and mRNAs to investigate the relationships of samples among two groups (sham and TBI) (Figures 1A–C). It could be found that the confidence ellipses of samples among sham and TBI groups were separate from eahc other based on the expression variances of lncRNAs, miRNAs, and mRNAs, indicating that the gene expression patterns were similar in the same group and significantly different between sham and TBI groups. Moreover, the sample clusterings were generated to investigate the similarity of samples among sham and TBI groups based on gene expressions of lncRNAs, miRNAs, and mRNAs (Figures 1D–F). On the other hand, it also could be found that the sample-to-sample distances were close in the same group and relatively far between sham and TBI groups. Finally, in order to confirm whether the lncRNA, miRNA, and mRNA expression data of samples between sham and TBI groups could be compared with each other, the box-whisker plots were used to show the degree of dispersion about the expression data distribution (Figures 1G–I). The distribution of lncRNA, miRNA, and mRNA expressions of samples between sham and TBI groups have no obvious difference, indicating that they were suitable for the gene expression comparison. Taken together, all these results illustrated the construction of the CCI model for TBI was repeatable and the reliability of these experiments was acceptable for further analysis.

### Differential expression of lncRNAs, miRNAs, and mRNAs in TBI

**FIGURE 2:**
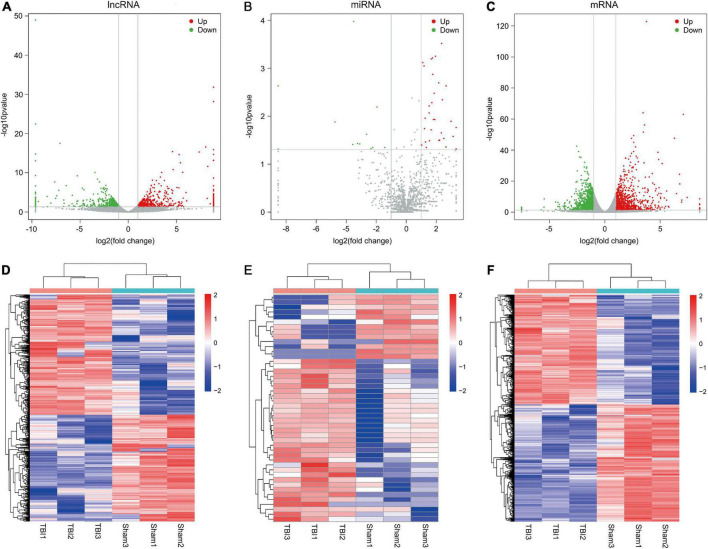
Differential expression profiles of lncRNAs, miRNAs, and mRNAs. **(A–C)** Volcano plots of differentially expressed lncRNAs, miRNAs, and mRNAs. Red dots indicates up-regulated genes and green dots indicates down-regulated genes. **(D–F)** Heatmaps of differentially expressed lncRNAs, miRNAs, and mRNAs. The color scale represents logarithmic transformation of normalized expression values.

The criteria for screening DE RNA (lncRNAs, miRNAs, and mRNAs) were *p*-value was less than 0.05 and fold change was greater than 2. DE lncRNAs, DE miRNAs, and DE mRNAs were shown using the volcano plot (Figures 2A–C) and heatmap (Figures 2D–F). Compared with the sham group, there were 939 DE lncRNAs (497 up-regulated and 442 down-regulated lncRNAs), 46 DE miRNAs (33 up-regulated and 13 down-regulated miRNAs), and 1,951 DE mRNAs (947 up-regulated and 1,004 down-regulated mRNAs). The top10 up-regulated and down-regulated lncRNAs/miRNAs/mRNAs based on the *p*-value were listed in Tables 1–3. Based on the *p*-value, the most up-regulated lncRNA, miRNA, and mRNA were *NRT006315.2* (fold change, inf; *p*-value, 1.44E-32), *novel478_mature* (fold change, 5.12; *p*-value, 3.04E-04), and *Spp1* (fold change, 13.55; *p*-value, 1.52E-123), respectively, and the most down-regulated lncRNA, miRNA, and mRNA were *NRT016522.2* (fold change, inf; *p*-value, 2.36E-49), *novel120_mature* (fold change, 0.09; *p*-value, 1.06E-04), and *Dclk3* (fold change, 0.17; *p*-value, 2.73E-43), respectively. The detailed information of all DE lncRNAs, DE miRNAs, and DE mRNAs was listed in Supplementary Tables 1–3.

**TABLE 1:** Top10 of differentially expressed up-regulated and down-regulated lncRNA.

lncRNA ID	Log_2_FC	*P*-value	Up/Down	Chromosome
*NRTT006315.2*	Inf	1.44E-32	Up	Chr10
*NRTT011512.2*	Inf	6.60E-29	Up	Chr16
*TCONS_00020887*	8.04	2.72E-17	Up	Chr2
*TCONS_00005661*	8.86	1.28E-16	Up	Chr10
*TCONS_00012967*	4.60	3.92E-16	Up	Chr15
*NRTT014638.2*	7.37	5.05E-16	Up	Chr19
*NRTT015732.2*	Inf	1.97E-15	Up	Chr2
*TCONS_00010614*	5.28	2.35E-15	Up	Chr13
*NRTT009739.2*	Inf	6.53E-14	Up	Chr14
*NRTT028463.2*	Inf	7.40E-14	Up	Chr8
*NRTT016522.2*	−Inf	2.36E-49	Down	Chr2
*NRTT013572.2*	−Inf	3.69E-23	Down	Chr18
*NRTT000902.2*	−7.09	3.00E-18	Down	ChrX
*TCONS_00022989*	−Inf	1.64E-15	Down	Chr3
*NRTT030122.2*	−Inf	8.61E-14	Down	Chr9
*NRTT000106.2*	−3.46	7.99E-11	Down	Chr1
*NRTT025964.2*	−Inf	5.01E-10	Down	Chr7
*NRTT016595.2*	−5.29	5.51E-10	Down	Chr2
*NRTT025229.2*	−3.19	4.00E-09	Down	Chr6
*NRTT007931.2*	−2.99	2.09E-08	Down	Chr12

Gene IDs with “NRT” prefix were known long non-coding RNAs which could be found from non-code website (www.noncode.org); Gene IDs with “TCONS” prefix were newly identified long non-coding RNAs in this study.

**TABLE 2:** Top10 of differentially expressed up-regulated and down-regulated miRNA.

miRNA ID	Log_2_FC	*P*-value	Up/Down	Sequence
*novel478_mature*	2.36	3.04E-04	Up	CGGGGGCCGGGCGGCGTC
*rno-miR-223-5p*	1.95	5.61E-04	Up	CGTGTATTTGACAAGCTGAGTTG
*novel86_star*	1.76	6.09E-04	Up	AGGGCTGGAGAGTTGGCTC
*novel776_mature*	1.67	6.38E-04	Up	GGGGGTGTAGCTCAGTGGTAGAGC
*rno-miR-451-5p*	1.11	7.61E-04	Up	AAACCGTTACCATTACTGAGTT
*rno-miR-155-5p*	1.19	8.98E-04	Up	TTAATGCTAATTGTGATAGGGGT
*novel896_mature*	1.77	1.33E-03	Up	AGGATGCTGCTGATGCTG
*rno-miR-147*	2.17	2.01E-03	Up	GTGTGCGGAAATGCTTCTGCTA
*novel214_mature*	2.66	2.34E-03	Up	CTCCCGGGGCCGAGGGGGC
*novel478_mature*	2.36	3.04E-04	Up	GGTTGGGGATTTAGCTCAGTGG
*novel120_mature*	−3.50	1.06E-04	Down	TCCTCTGTCCCCACCCATCCCCAGG
*rno-miR-6321*	−8.54	2.15E-04	Down	TACTGCAGTGAGTTCTATGAAGC
*rno-miR-292-5p*	−Inf	2.33E-03	Down	ACTCAAACTGGGGGCTCTTTTG
*rno-miR-96-5p*	−1.94	0.01	Down	TTTGGCACTAGCACATTTTTGCT
*rno-miR-293-5p*	−4.75	0.01	Down	ACTCAAACTGTGTGACACTTT
*novel233_mature*	−2.64	0.02	Down	TGATGCCACCAACCCTCCCACAGA
*novel625_mature*	−3.24	0.04	Down	ATCTCGGTGTTTGGGCTG
*novel259_star*	−3.09	0.04	Down	CTTGCCCCAACCTGTTGCCAGC
*novel387_mature*	−3.56	0.04	Down	AGTGTCTATGCTGTTCTCATTT
*rno-miR-216a-5p*	−2.23	0.04	Down	TAATCTCAGCTGGCAACTGTGA

Gene IDs with “rno-miR” prefix were known microRNAs which could be found from miRBase website (http://www.mirbase.org/); Gene IDs with “novel” prefix were newly identified microRNAs in this study.

**TABLE 3:** Top10 of differentially expressed up-regulated and down-regulated mRNA.

mRNA ID	Log_2_FC	*P*-value	Up/Down	Annotation
*Spp1*	3.76	1.52E-123	Up	Secreted phosphoprotein 1
*Gldn*	3.45	9.53E-65	Up	Gliomedin
*Helt*	7.08	1.03E-63	Up	Helt bHLH transcription factor
*Ccl2*	3.57	6.63E-57	Up	C-C motif chemokine ligand 2
*LOC303140*	3.48	2.57E-52	Up	Up-regulator of carnitine transporter, OCTN2
*Runx1*	2.61	4.96E-50	Up	Runt-related transcription factor 1
*Fam83g*	6.28	2.29E-48	Up	Family with sequence similarity 83, member G
*Tgfb2*	2.41	3.05E-48	Up	Transforming growth factor, beta 2
*Cyp1b1*	2.61	1.08E-45	Up	Cytochrome P450, family 1, subfamily, polypeptide 1
*Cd14*	2.77	9.41E-43	Up	CD14 molecule
*Dclk3*	−2.53	2.73E-43	Down	Doublecortin-like kinase 3
*Akap5*	2.36	7.84E-40	Down	A-kinase anchoring protein 5
*Fn3k*	2.27	2.66E-36	Down	Fructosamine 3 kinase
*Ankrd34c*	2.15	4.85E-36	Down	Ankyrin repeat domain 34C
*Cdh12*	−1.82	9.34E-33	Down	Cadherin 12
*Plch2*	1.85	8.41E-30	Down	Phospholipase C, eta 2
*Mal*	−1.62	8.57E-30	Down	mal, T-cell differentiation protein
*Car2*	1.64	3.80E-29	Down	Carbonic anhydrase 2
*Shroom2*	1.66	4.66E-29	Down	Shroom family member 2
*Tacr3*	2.48	2.46E-27	Down	Tachykinin receptor 3

### GO enrichment and KEGG pathway analyses of DE mRNAs in TBI

**FIGURE 3:**
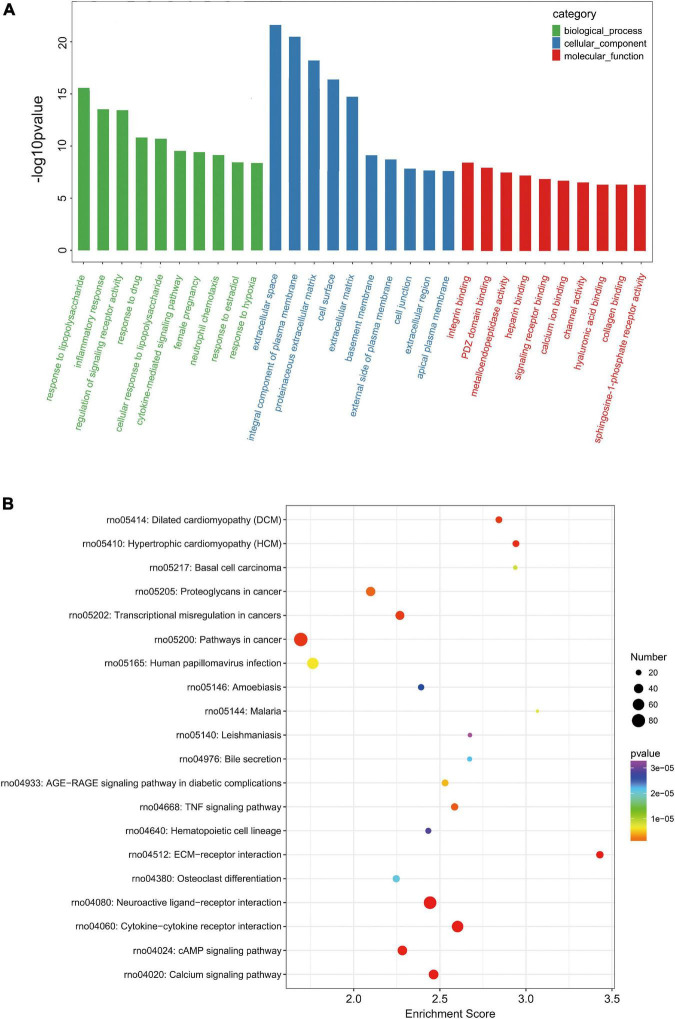
Gene ontology (GO) and KEGG pathway analysis of DE mRNAs. **(A)** Top10 GO categories of biological processes, cellular components, and molecular functions. The GO categories are arranged according to the *p*-value. **(B)** Top20 enriched KEGG pathway of DE mRNAs. The bubble size indicates the number of DE mRNAs and the color scale indicates the *p*-value.

In order to further analyze the DE mRNAs related to TBI pathophysiology, GO enrichment and KEGG pathway analyses were performed. The GO enrichment analysis revealed that these 1,951 DE mRNAs in TBI are mainly related to response to lipopolysaccharide (biological_process), extracellular space (cellular_component), and integrin binding (molecular_function) (Figure 3A). The KEGG pathway analysis showed the top20 enriched pathways in these 1,951 DE mRNAs in TBI. Of them, pathways in cancer, neuroactive ligand-receptor interaction, and cytokine-cytokine receptor interaction were the most enriched pathways (Figure 3B). Moreover, the complete list with significantly (*p*-value < 0.05) enriched pathways was listed in Supplementary Table 4. It was found that these KEGG pathway closely related to apoptosis, inflammation, angiogenesis, and neurological functions, including calcium signaling pathway, TNF signaling pathway, Jak-STAT signaling pathway, PI3K-Akt signaling pathway, HIF-1 signaling pathway, and so on.

### Protein-protein interaction network of hub DE mRNAs in TBI

**FIGURE 4:**
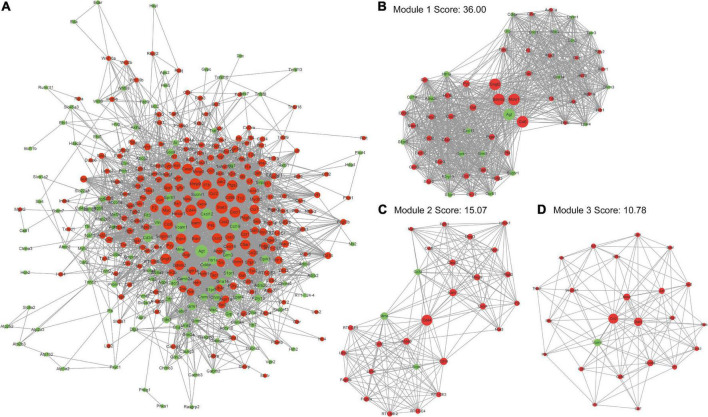
Protein interaction network analyses of proteins encoding by DE mRNAs. **(A)** Protein-protein interaction (PPI) network of DE mRNA. **(B–D)** Three sub-network clusters obtained by MCODE clustering analysis of PPI network (node degree >10). The size of node represents the degree in the PPI network. The red color represents up-regulated node and the green color represents down-regulated node in the PPI network.

To fully explore the key genes related to pathophysiological development of TBI, the PPI network of top20 enriched pathways related DE mRNAs was built on the STRING website. It could be obtained 3,611 protein interactions of 353 DE mRNAs, of which, 208 DE mRNAs were up-regulated and 145 DE mRNAs were down-regulated (Figure 4A). Then, cytoHubba plug-in of Cytoscape software was used to screen out key proteins in the PPI network according to the result of node degree calculation. The proteins with higher degree level were more inclined to the key proteins in TBI. Moreover, MCODE plug-in of Cytoscape software was used to perform a clustering analysis based on the PPI network. Three sub-network modules with scores 36.00, 15.07, and 10.78 were displayed in Figures 4B–D, respectively. Module 1 contained 1080 PPI pairs corresponding to 61 mRNAs (39 up-regulated genes and 22 down-regulated genes) (Figure 4B). *Agt*, *Ccl6*, *Bdkrb2*, *Mchr1*, and *Gngt2* acted as bridges to connect two groups of genes, indicating manipulation of these five proteins had more impacts on the genes in these two groups. Module 2 contained 211 PPI pairs corresponding to 29 genes (26 up-regulated genes and three down-regulated genes) (Figure 4C). *Cd44* acted as a bridge to connect two groups of genes, indicating intervention of *Cd44* would be likely to influence the functions of the genes in these two groups. Module 3 contained 124 PPI pairs corresponding to 24 genes (23 up-regulated genes and one down gene) (Figure 4D). The roles of *Ccl2*, *Stat3*, and *Vcam1* were important than any other proteins in the module. To sum up, these proteins were more effective intervention targets to alleviate the secondary injury caused by TBI.

### Construction of the lncRNA associated ceRNA network in TBI

**FIGURE 5:**
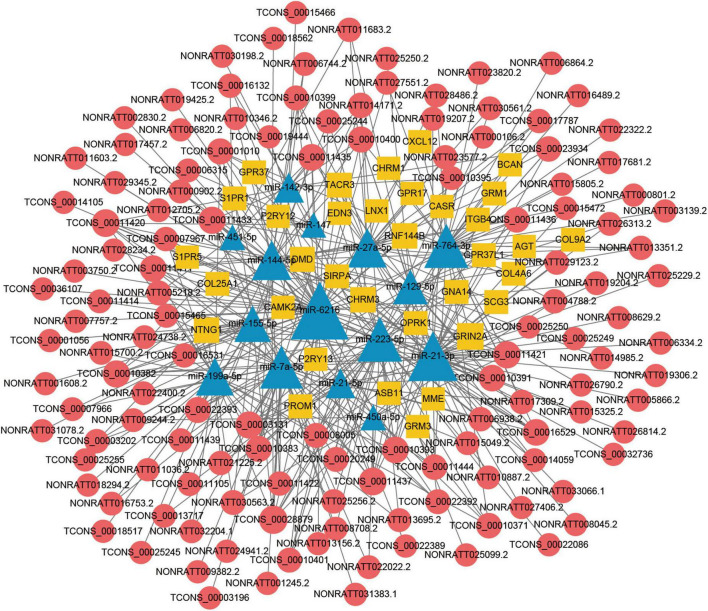
Long non-coding RNA (LncRNA) associated ceRNA networks of up-regulated miRNA in TBI. Yellow rhombuses represent mRNA nodes, red rounds represent lncRNA, and blue triangles represent miRNA nodes. The size of the node represents the degree in the ceRNA network.

**FIGURE 6:**
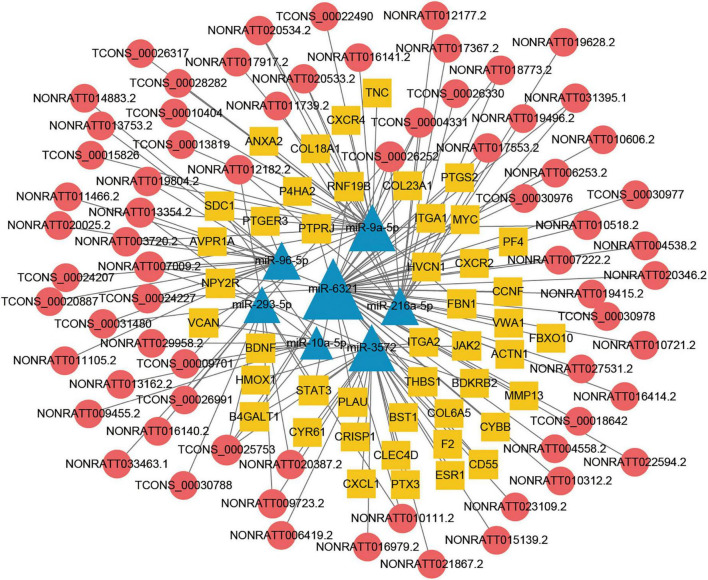
Long non-coding RNA (LncRNA) associated ceRNA networks of down-regulated miRNA in TBI. Yellow rhombuses represent mRNA nodes, red rounds represent lncRNA, and blue triangles represent miRNA nodes. The size of the node represents the degree in the ceRNA network.

**FIGURE 7:**
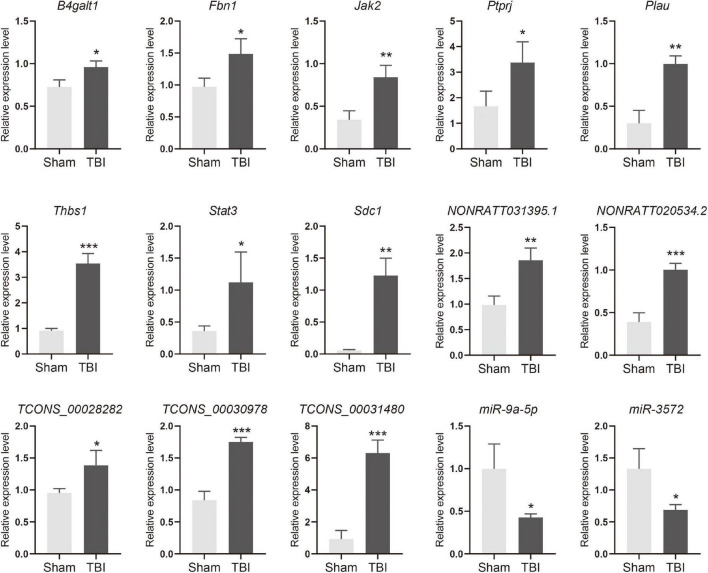
Quantitative RT-PCR verifications of upregulated mRNA, lncRNA, and downregulated miRNA in ceRNA networks. *p < 0.05, **p < 0.01 and ***p < 0.001.

The lncRNA-miRNA-mRNA ceRNA networks were constructed on the 79 hub mRNAs identified by PPI network (degree >10). The ceRNA networks were under following criteria, the expressions of lncRNA and mRNA were with the same trend, that is they were both up-regulated or down-regulated. Because lncRNA bind to miRNA in the cytoplasm, lncRNA predicted to be no in the cytoplasm were excluded, As a result, miRNA-targeted pairs (miRNAs–mRNAs and miRNAs–lncRNAs) including 201 lncRNAs, 22 miRNAs, and 79 mRNAs were screened out and were used to construct the lncRNA associated ceRNA networks (Supplementary Table 5). Two ceRNA networks were successfully built, including up-regulated miRNA network with 132 lncRNAs, 15 miRNAs, and 34 mRNAs and a down-regulated miRNA network with 69 lncRNAs, 7 miRNAs, and 45 mRNAs (Figures 5, 6). To validate the expression of genes included in the ceRNA network. miRNA, lncRNA, mRNA was chosen to conduct the QRT-PCR experiment. Primers were shown in Supplementary Table 6. The results of QRT-PCR showed that the expression changes of most genes were in general agreement with the data of RNA-seq analysis. mRNA *B4galt1*, *Fbn1*, *Jak2*, *Ptprj*, *Plau*, *Thbs1*, *Stat3*, and *Sdc1* were up-regulated after TBI. lncRNA *NONRATT031395.1*, *NONRATT020534.2*, *TCONS_00028282*, *TCONS_00030978*, and *TCONS_00031480* were up-regulated after TBI. miRNA *miR-9a-5p*, and *miR-3572* were down-regulated after TBI (Figure 7). To sum up, these verified lncRNAs were candidate targets that could be selected for future work.

## Discussion

Recently, a growing body of studies revealed that many types of ncRNAs including lncRNAs, miRNAs, and circRNAs played important roles in the initiation and progression of secondary injuries following TBI (Chandran et al., 2017). The lncRNAs *NEAT1* and *MALAT1* were beneficial to reduce adverse outcomes following TBI in transplantation of human adipose-derived stem cells (hADSCs) and bexarotene could up-regulate *NEAT1* to get better functional recovery by inhibiting apoptosis and inflammation caused by TBI (Tajiri et al., 2014; Zhong et al., 2017). Previous studies reported that a large number of lncRNAs and mRNAs were DE after TBI and lncRNAs were likely to perform their functional roles in TBI through regulating the expressions of mRNAs related to inflammation, apoptosis, and neuronal and vascular networks (Ge et al., 2014; Pan et al., 2017). Actually, the main ways of lncRNAs on regulating the expressions of mRNAs through influencing the expression and/or chromatin state of nearby genes and interacting with proteins and/or other RNA molecules such as miRNAs (Marchese et al., 2017). Recent study have revealed that downregulation of miR-491-5p promotes neovascularization after TBI (Tang et al., 2022b), and lncRNA-AK046375 enhances MT2 expression by sequestering miR-491-5p, ultimately strengthening antioxidant activity, which ameliorates neurological deficits post-TBI (Tang et al., 2022a). These studies show the prospect of ceRNA regulation mechanism in TBI research. In this study, we performed RNA sequencing of lncRNAs, miRNAs, and mRNAs in CCI model of TBI (Figures 1, 2) and we constructed lncRNA associated ceRNA networks based on bioinformatics prediction of lncRNA–miRNA and miRNA–mRNA interaction pairs (Figure 5).

To date, the effective drugs for the treatments of patients with TBI were still lacking and several candidates such as nicotinamide, simvastatin, cyclosporine, levetiracetam, and erythropoietin were tested by the Operation Brain Trauma Therapy (OBTT) consortium in animals (Kochanek et al., 2016). Compared with the chemotherapeutic agent, lncRNA associated ceRNA would provide an alternative selection to be intervention targets in TBI. For example, the up-regulation of lncRNA could compete for sponging increased amount of miRNAs to reduce post-transcriptional silencing or degradation of target RNAs. Most lncRNAs contain 1–10 MREs and 50% of miRNAs target 1–400 mRNAs (Cai and Wan, 2018). Under certain conditions, single lncRNA perturbation would influence many downstream target mRNAs through miRNA mediators which leading to the amplification of lncRNA effects on genes related to downstream signaling cascades. Encouragingly, some miRNAs that had been reported in TBI pathogenesis were included in the ceRNA networks, such as *miR-9a-5p*, overexpression of miR-9a-5p ameliorates NLRP1 inflammasome-mediated ischemic injury in rats following ischemic stroke (Cao et al., 2020). And it is down-regulated in our research. Besides, there were many pieces of evidences supporting ceRNA regulation in neurodegenerative disorders mediated by miRNAs and it was believed that TBI shared common molecular mechanisms with neurodegenerative diseases. Based on the above evidence, we had reasons to speculate that the DE lncRNAs identified in this study could compete with miRNAs mentioned above to reduce secondary injuries following TBI.

However because of the complexity of the lncRNA associated ceRNA networks, the selection of ideal lncRNAs that were capable of alleviating secondary injuries following TBI through manipulating the expressions of suitable mRNAs was still to be elucidated. Thus, GO enrichment, KEGG pathway, and PPI network analyses were used to filtered these key DE mRNAs related to pathophysiological development of TBI (Figures 3, 4). Moreover, the key miRNA mediators with a large number of target mRNAs were well selections to minimize the list of intervention targets. For example, *miR-3572* could target 20 mRNAs including a large number of genes involved in the pathophysiological development of TBI, such as *Esr1*, *Bdnf*, *Jak2*, *Vcan*, *and Stat3*. In addition, our study used a *P*-value rather than the widely accepted padj. In our study data, FC > 2 was maintained, and 1,951 differential genes were found when mRNA was used at *p* < 0.05, and 1,720 differential genes were found when padj <0.05. lncRNA with *p* < 0.05 showed 939 differential genes, padj with 156 differential genes. miRNA with *p* < 0.05 showed 46 differential genes, padj <0.05 showed 0 differential genes. However, when we specifically looked at the relative expression levels of those genes excluded by padj, it was found that the relative expression levels of many genes between the two groups were significantly different, for example, the relative expression levels of NONRATT020534.2, three samples in sham group were 1.25, 0.55, 1.14, and that of the TBI group were 5.45, 3.59, 2.51. Calculated *p*-value = 0.0012 < 0.05, but padj = 0.093 > 0.05. And, there were differences between the TBI group and the sham group in the PCR validation experiment, *p* = 0.0013 < 0.05. In addition, miR-9a-5p was significantly down-regulated in the rat MCAO model (Wang et al., 2018; Cao et al., 2020). In our sequencing data, it was excluded by padj <0.05. But it was also significantly down-regulated by QRT-PCR verification. So we think that padj will cause us to miss a lot of important genes due to the inevitable limitations of statistical tools. Of course, the use of *P*-values instead of padj also means a much higher false positive rate. Thus, though our study constructed a ceRNA regulatory network after TBI, further research still requires lots of validation experiments, including QRT-PCR and Dual-Luciferase Reporter Assay. And whether these candidate gene target suitable for further investigation in TBI treatment require more *in vivo* evidences.

## Conclusion

Our study performed whole transcriptome resequencing about lncRNAs, miRNAs, and mRNAs in the CCI model of TBI rats. The ceRNA regulatory network of 302 key genes was constructed based on the KEGG pathway and PPI network analyses. These findings would provide novel understandings about the molecular mechanisms in the pathophysiological processes of TBI. Even more, these novel ceRNA networks could be potential therapeutic targets in TBI.

## Data availability statement

The datasets presented in this study can be found in online repositories. The names of the repository/repositories and accession number(s) can be found in the public database SRA (accession: PRJNA898136).

## Ethics statement

This animal study was reviewed and approved by the Institutional Animal Care and Use Committee of Wuhan University (IACUC: 2019010).

## Author contributions

YZ and HM designed the research. JY, RL, and PW conducted the experiments. JY and ZL finished the data analysis and manuscript. HM instructed the writing and figures creation. All authors read and approved the manuscript for publish.

## Footnotes

1
http://www.mirbase.org/

2
http://www.geneontology.org

3
https://www.genome.jp/kegg

4
http://frodo.wi.mit.edu/primer3/
